# Supplementary material for: Quality of Life Scores Remained Different among the Genotypic Groups of Patients with Suspected Hemochromatosis, Even after Treatment Period
Source: Genes (Basel). 2022 Jan 10;13(1):118. doi: 10.3390/genes13010118 (PMC8774363; doi:10.3390/genes13010118)
Supplement: Supplementary file 1 [file genes-13-00118-s001.zip › genes-1515792-suplementary.pdf]

Additional File 1: Table S1

Supplementary Table S1. Comparison of mean (±standard deviation) values of the SF-36 domains according with initial and final phases among patient groups

Variables		Group 1[a] n = 23		Group 2[b] n = 42	
**SF-36 domains**	**Follow-up phases**	**Mean ± SD**	**p value**	**Mean ± SD**	**p value**
**Physical functions**	Initial	81 ± 23	0.74	89 ± 15	0.20
	Final	84 ± 24		82 ± 22	
**Role-physical**	Initial	74 ± 42	0.34	86 ± 30	0.31
	Final	74 ± 43		73 ± 36	
**Bodily pain**	Initial	70 ± 21	0.19	78 ± 22	0.60
	Final	64 ± 30		74 ± 24	
**General health perception**	Initial	66 ± 21	0.34	66 ± 15	0.30
	Final	58 ± 21		69 ± 21	
**Vita lity**	Initial	54 ± 26	0.92	67 ± 19	0.60
	Final	56 ± 24		67 ± 21	
**Social functioning**	Initial	70 ± 32	0.64	87 ± 16	0.65
	Final	68 ± 31		87 ± 22	
**Role-emotional**	Initial	75 ± 37	0.02	82 ± 31	0.31
	Final	57 ± 45		83 ± 30	
**Mental health**	Initial	66 ± 23	0.94	74 ± 18	0.30
	Final	69 ± 22		77 ± 21	

[a]Group 1: 23 patients with primary iron overload and homozygosity for the p.Cys282Tyr mutation. [b]Group 2:

42 patients with primary iron overload and other genotypes: compound heterozygosity for the p.Cys282Tyr/p.His63Asp (n = 10), heterozygosity for the p.Cys282Tyr (n = 4), homozygosity (n = 10) or heterozygosity (n = 8) for the p.His63Asp, or absence of p.Cys282Tyr and p.His63Asp (n = 10).

Additional File 2: Table S2

Supplementary Table S2. Comparison of serum ferritin (ng/mL) values and the SF-36 domainsaccording with initial and final phases in overall patient group

		Serum ferritin initial phase		Serum ferritin finalphase
**Physical functions (initial phase,final phase)**	r	-0.212	R	-0.352
	p	0.121	P	0.008
**Role-physical (initial phase, finalphase)**	r	-0.060	R	-0.133
	p	0.661	P	0.332
**Bodily pain (initial phase, finalphase)**	r	-0.141	R	-0.038
	p	0.305	P	0.784
**General health perception(initial phase, final phase)**	r	0.008	R	-0.127
	p	0.953	P	0.354
**Vitality (initial phase, finalphase)**	r	-0.024	R	-0.211
	p	0.864	P	0.121
**Social functioning (initial phase,final phase)**	r	-0.173	R	-0.200
	p	0.207	P	0.142
**Role-emotional (initial phase,final phase)**	r	0.157	R	-0.094
	p	0.253	P	0.495
**Mental health (initial phase,final phase)**	r	0.064	R	0.110
	p	0.643	P	0.424
